# Identification and validation of a glycolysis-related gene signature for depicting clinical characteristics and its relationship with tumor immunity in patients with colon cancer

**DOI:** 10.18632/aging.204226

**Published:** 2022-08-13

**Authors:** Gang Liu, Xiaoyang Wu, Jian Chen

**Affiliations:** 1 Suzhou Medical College of Soochow University, Suzhou 215300, Jiangsu Province, China; 2 Department of General Surgery, Affiliated Kunshan Hospital of Jiangsu University, Suzhou 215300, Jiangsu Province, China

**Keywords:** colon cancer, glycolysis, patient prognosis, clinical features, tumor immunity

## Abstract

Colon cancer (CC) is one of the most common gastrointestinal malignant tumors with a high mortality rate. Glycolysis is an important pathway for tumors to obtain energy. However, its role in CC remains largely unknown. In present study, we analyzed glycolysis-related gene expression to depict clinical characteristics and its relationship with tumor immunity in CC to find potential target treatments. A prognostic model based on 13 glycolysis-related genes was established by univariate and multivariate Cox regression analyses. The efficacy of the gene model was tested via survival analysis, receiver operating characteristic analysis, and principal component analysis. Furthermore, our findings revealed and validated 13 glycolysis-related genes (*NUP107*, *SEC13*, *ALDH7A1*, *ALG1*, *CHPF*, *FAM162A*, *FBP2*, *GALK1*, *IDH1*, *TGFA*, *VLDLR*, *XYLT2*, and *OGDHL*), which constituted a prognostic prediction model. The model exhibited clinical implication potential, had a relatively high accuracy, and was closely associated with the patients’ clinical features. In particular, the tumor stage could be clearly distinguished by glycolysis-related gene signatures. Finally, a significant difference between glycolysis-related gene colon cancer immunity and sensitive immune drugs was observed. Our glycolysis-related gene model could provide the basis for potential early individualized treatment. The 13 glycolysis-related gene signature was a reliable predictive tool for the prognosis of colon cancer. Our findings could help patients select targets for individualized treatment and immunotherapy strategies. The study findings advance our understanding of the potential mechanism of glycolysis in colon cancer.

## INTRODUCTION

Colon cancer (CC) is the third most common gastrointestinal malignant tumor derived from glandular epithelial cells, with a high mortality and increasing incidence [1]. Despite recent improvements in the diagnosis and treatment of CC, including surgery, radiotherapy, chemotherapy, neoadjuvant chemotherapy, and immunotherapy, the 5-year relative survival rate remains poor, and distant metastasis remains the main cause of CC-associated death [2, 3]. Therefore, there is an urgent need to explore potentially useful diagnostic and prognostic biomarkers for CC patients.

Glycometabolism, a vital pathway in living cells, differs significantly between normal cells and tumor cells. The transformation of glucose utilization from oxidative phosphorylation to glycolysis is now regarded as a major feature of cancer. This kind of change in energy metabolism is regulated by genetic changes and tumor microenvironment pressure, causing an increase in the cell proliferation rate and conferring resistance on cells [4]. The activation of glycolysis can affect other phenotypic processes, such as epithelial-to-mesenchymal transition (EMT) [5], which is closely associated with glycolysis. Zhao et al. reported that activated glycolysis promotes pancreatic cancer cell stemness and EMT [6]. Numerous studies have reported that the disturbance of immune regulation is closely associated with cancer, whereas the relationship between tumor immunity and CC glycolysis has not been explored. Hence, understanding the underlying interplay between glycolysis and cancer progression is a critical goal in cancer research that can clarify cancer pathogenesis and facilitate the development of effective therapeutic approaches.

In our study, gene set enrichment analysis (GSEA) of glycolysis-related pathways was conducted using 566 CC and 19 normal control array data from the Gene Expression Omnibus (GEO) to identify differentially expressed glycolysis genes in CC patients. Then, following Cox regression analysis, the patients were classified into low- and high-risk groups according to glycolysis-related expression and risk score. A signature consisting of 13 genes was constructed, which was closely associated with poor overall survival (OS), older age, high tumor grades, high EMT, different immune cell infiltration and targeted sensitive immune drugs. The risk score could serve as an independent pathological factor. Finally, the expression of the 13 signature genes was validated in our patient samples. Overall, our data revealed a strong association between glycolysis metabolism and clinical prognosis in CC.

## RESULTS

### Clinical characteristics of CC patients

Overall, 566 CC and 19 normal control data stored on the Affymetrix Human Genome U133 Plus 2.0 Array platform were downloaded from a GEO dataset. The clinical characteristics of the samples, including sex, age, survival status, survival time, clinical TNM stage, clinical T stage, clinical N stage, and clinical M stage, are shown in Table 1.

**Table 1:** Clinical information of CC patients.

**Variables**	**GSE39582 Patients (566)**	**TCGA Patients (385)**
**Fustat**		
Alive	370	314
Death	191	71
unknown	5	
**Age (year)**		
<=65	222	159
>65	344	226
**Gender**		
Male	256	205
Female	310	180
**TNM Stage**		
I	33	66
II	264	151
III	205	103
IV	60	54
0	4	11
**T stage**		
T1	11	9
T2	45	68
T3	367	263
T4	119	44
Tis	3	1
T0	1	0
unknown	20	0
**M stage**		
M0	482	286
MI	61	54
MX	0	39
unknown	23	6
**N stage**		
N0	302	231
N1	134	88
N2	98	66
N3	6	0
unknown	26	0

### Differentially expressed glycolysis genes between the CC and normal control samples

**Figure 1:**
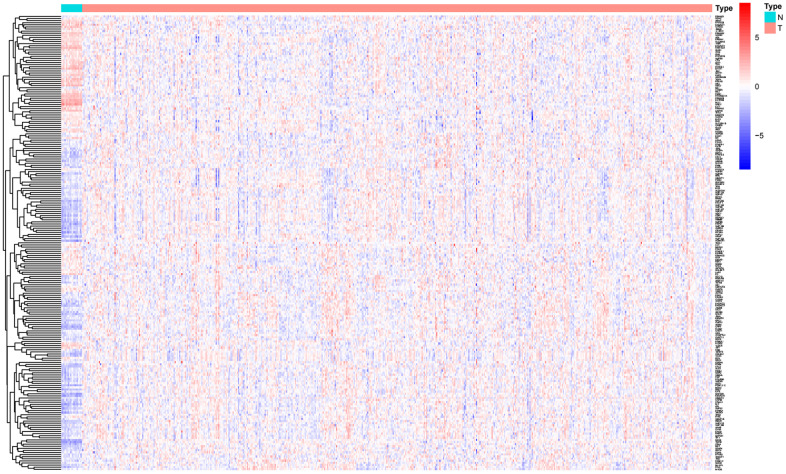
Significantly differentially expressed glycolysis-related genes between CC and normal controls.

Six glycolysis-related gene sets were obtained from the Molecular Signatures Database: REACTOME_GLYCOLYSIS, WP_COMPUTATIONAL_MODEL_OF_AEROBIC_GLYCOLYSIS, WP_GLYCOLYSIS_IN_SENESCENCE, REACTOME_REGULATION_OF_GLYCOLYSIS_BY_FRUCTOSE_2_6_BISPHOSPHATE_METABOLISM, HALLMARK_GLYCOLYSIS, and GO_GLYCOLYTIC_PROCESS. A total of 301 glycolysis-related genes were selected from the six gene sets for additional research, and 217 glycolysis-related genes were found to be significantly and differentially expressed in the CC samples compared with the controls (Figure 1).

### Construction of the glycolysis-related gene prognostic prediction model for CC patients

**Figure 2:**
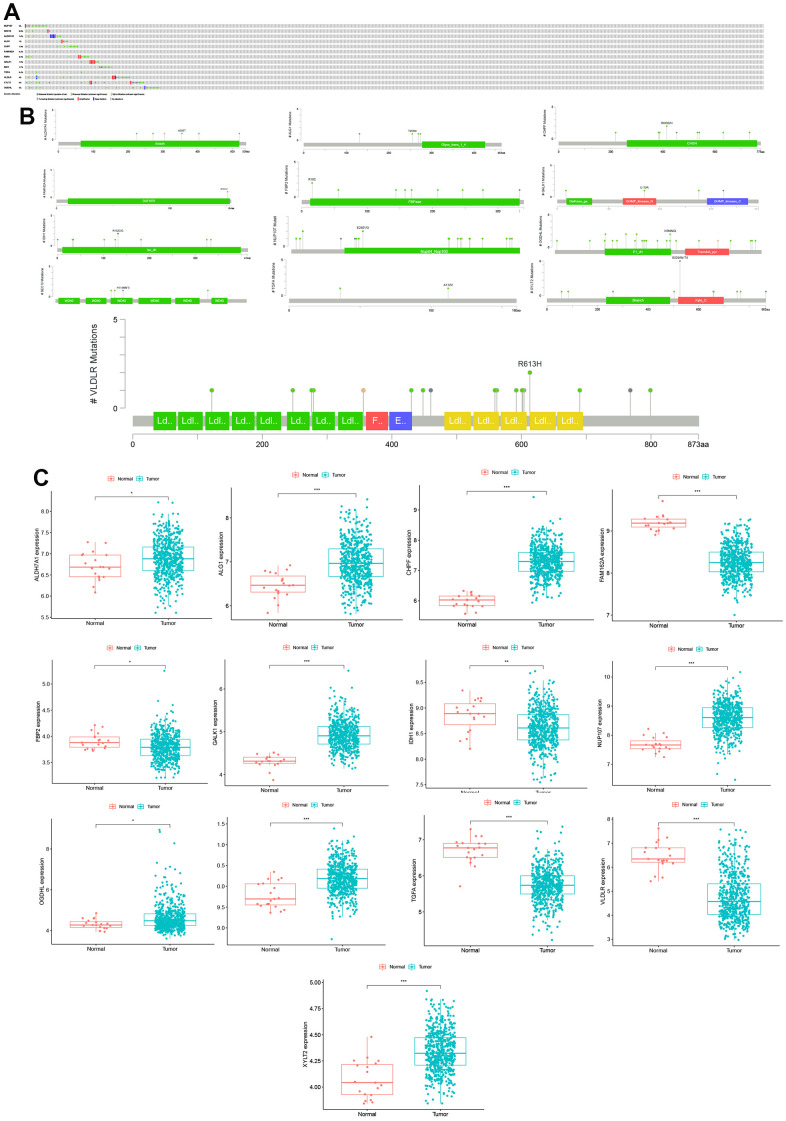
**Mutations and expression levels of model genes in CC.** (**A**) Mutation frequency of model genes. (**B**) Model gene-specific mutation domains. (**C**) Model gene expression between CC and normal control samples.

UniCox and multiCox regression analyses were performed on the differentially expressed glycolysis-related genes to construct a novel biomarker model for predicting the prognosis of CC patients. Thirteen risk genes (*NUP107*, *SEC13*, *ALDH7A1*, *ALG1*, *CHPF*, *FAM162A*, *FBP2*, *GALK1*, *IDH1*, *TGFA*, *VLDLR*, *XYLT2*, and *OGDHL*) were identified (P < 0.05, Table 2). Then, the prognostic gene model based on the 13 glycolysis-related genes was used to divide patients into low- and high-risk groups as follows: Risk score = (-0.29×NUP107 Expression + [-0.35×SEC13 Expression] + 0.30×ALDH7A1 Expression + [-0.29×ALG1 Expression] + 0.45×CHPF Expression + [-0.46×FAM162A Expression] + 0.91×FBP2 Expression + [-0.65×GALK1 Expression] + [-0.34×IDH1 Expression] + 0.36×TGFA Expression + 0.18×VLDLR Expression + [-1.28×XYLT2 Expression] + [-0.41×OGDHL Expression]). The alterations in the 13 genes in the CC tissues were analyzed, and the results showed that the alteration rates in *NUP107*, *SEC13*, *ALDH7A1*, *ALG1*, *CHPF*, *FAM162A*, *FBP2*, *GALK1*, *IDH1*, *TGFA*, *VLDLR*, *XYLT2*, and *OGDHL* were 3%, 0.8%, 1.9%, 1%, 1.9%, 0.2%, 2.3%, 1.5%, 1.7%, 0.4%, 4%, 4%, and 4%, respectively (Figure 2A). Many mutations occurred in the gene domains, and the specific mutation sites are presented in Figure 2B. Finally, the expression of the 13 genes in the CC patient and normal tissues was further analyzed. *NUP107*, *SEC13*, *ALDH7A1*, *ALG1*, *CHPF*, *GALK1*, *XYLT2*, and *OGDHL* were highly expressed in CC tissues, while *FAM162A*, *FBP2*, *IDH1*, *TGFA*, and *VLDLR* were downregulated (P < 0.05, Figure 2C).

**Table 2:** Thirteen genes were selected via uniCox regression analysis.

**ID**	**HR**	**HR.95 L**	**HR.95H**	**CoxPvalue**
NUP107	0.64	0.49	0.85	0.00
SEC13	0.64	0.43	0.95	0.03
ALDH7A1	0.70	0.50	0.97	0.03
ALG1	0.60	0.44	0.82	0.00
CHPF	1.38	1.03	1.85	0.03
FAM162A	0.59	0.40	0.88	0.01
FBP2	2.41	1.33	4.38	0.00
GALK1	0.38	0.24	0.60	0.00
IDH1	0.54	0.36	0.80	0.00
TGFA	1.75	1.26	2.43	0.00
VLDLR	1.17	1.02	1.35	0.03
XYLT2	0.28	0.13	0.61	0.00
OGDHL	0.56	0.41	0.75	0.00

### Efficacy of the risk score in CC patients

**Figure 3:**
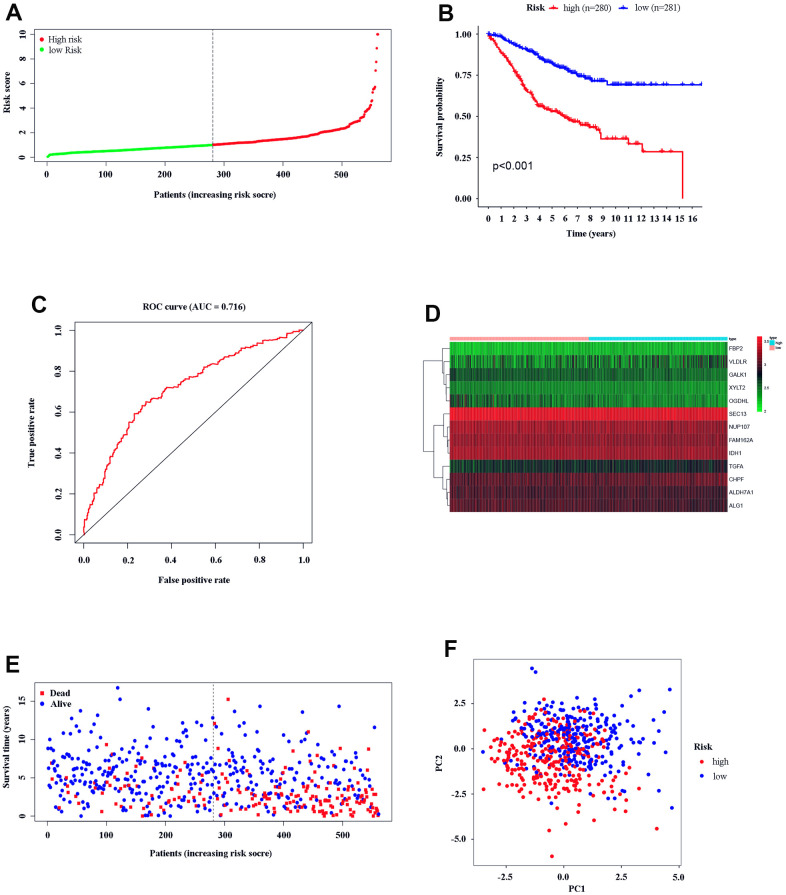
**Text efficacy of the gene model in CC patients.** (**A**) CC patient distribution according to the median risk score. (**B**) Kaplan–Meier analysis between the low- and high-risk groups. (**C**) ROC analysis of the risk score. (**D**) Model gene expression in the low- and high-risk groups. (**E**) The relationship between patient survival status and risk score. (**F**) PCA between the low- and high-risk groups.

Based on the gene model, 280 CC patients and 281 CC patients were classified into high- and low-risk groups by the median risk score (Figure 3A). KM analysis was carried out between the low- and high-risk groups, and the high-risk group had a significantly poorer prognosis (P < 0.05, Figure 3B). ROC analysis was carried out, and the AUC was calculated to be 0.716, which showed a good prediction effect (Figure 3C). The expression of the 13 genes was calculated; TGFA FBP2, CHPF, and VLDLR were significantly higher in the high-risk group, and NUP107, OGDHL, SEC13, IDH1, GALK1, FAM162A, ALG1, and XYLT2 ALDH7A1 were downregulated (P < 0.05, Figure 3D). The risk plot indicates that the high-risk group was closely related to poor prognosis (Figure 3E). PCA showed that high- and low-risk groups could be significantly distinguished using our model, which reduced the dimension of the expression of multiple genes (Figure 3F). All the analysis results indicated that our gene model had good efficacy based on the risk score.

### Independent risk factor for risk score in CC patients and its relationship with clinical characteristics of the patients

**Figure 4:**
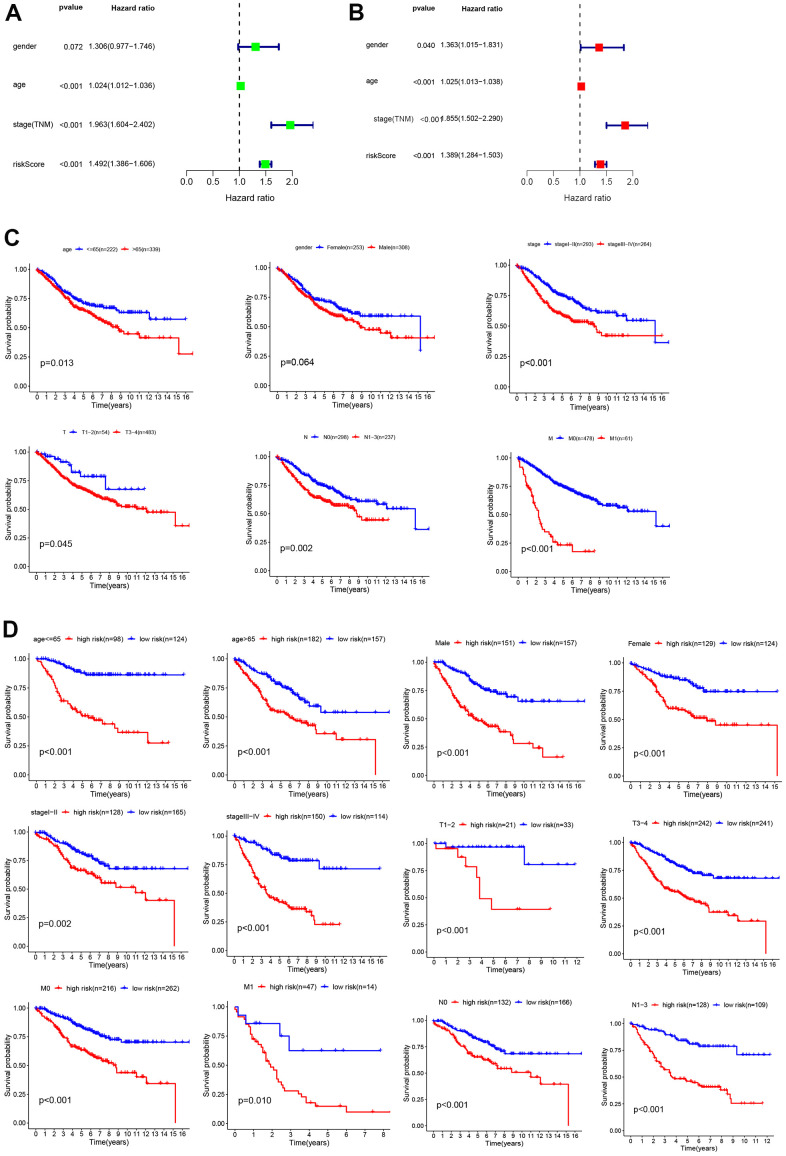
**Analysis of risk factors.** Univariate (**A**) and multivariate Cox regression analyses (**B**) of the relationship between risk core and other clinical features. (**C**) Kaplan–Meier analysis of clinical features, including age, sex, TNM stage, T stage, N stage, and M stage. (**D**) Kaplan–Meier analysis of clinical features in low- and high-risk subgroups.

Univariate and multivariate independent prognostic analyses were carried out to identify independent prognostic factors, including age, sex, TNM stage, and risk score. The results showed that age, TNM stage, and risk score were independent prognostic factors in CC patients, and they were all positively correlated with poor survival prognosis (P < 0.05, Figure 4A, 4B). For a more accurate analysis of the relationship between risk factors and patient OS, a series of Kaplan–Meier curve analyses was carried out. The results revealed that an age of > 65 years, TNM III–IV, T3-4, N1–3 and M1 were positively related to poor OS (P < 0.05, Figure 4C). The relationship between patients’ clinical characteristics and OS in the low- and high-risk groups was determined, and high-risk patients were positively associated with a poor OS when age ≤ 65 years, age>65 years, female sex, male sex, TNM I–II, TNM III–IV, T1–2, T3–4, M0, M1, N0, and N1-3 subgroups (P < 0.05, Figure 4D). These results demonstrate that our glycolysis-related gene model could distinguish clinical features and show the reliability of our glycolysis-related gene model in CC.

### Role of EMT in a high glycolysis tumor environment

**Figure 5:**
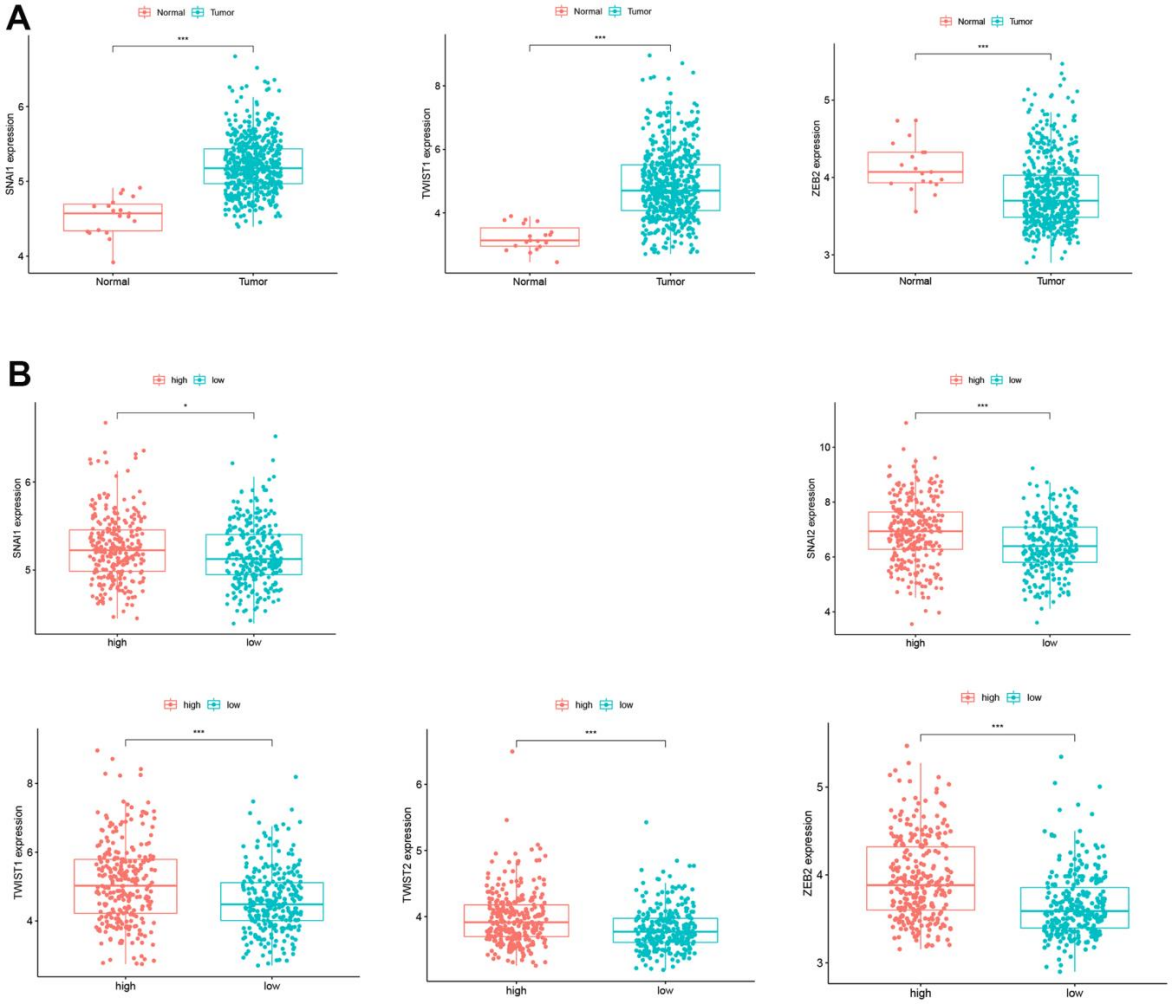
**Expression of EMT biomarkers.** (**A**) SNAI1 and TWIST1 expression in CC patients and normal controls. (**B**) SNAI1, SNAI2, TWIST1, TWIST2, and ZEB2 expression in the low- and high-risk groups.

Studies have demonstrated that glycolysis can promote EMT in cancers. To explore the role of EMT in CC, we analyzed the expression of EMT biomarkers (SNAI1, SNAI2, TWIST1, TWIST2, and ZEB2) in our study. SNAI1 and TWIST1 showed significantly higher expression in CC tissues (P < 0.05, Figure 5A). Moreover, SNAI1, SNAI2, TWIST1, TWIST2, and ZEB2 expression was upregulated in the high-risk group (P < 0.05, Figure 5B). This suggests that EMT plays a role in the high-glycolytic tumor environment of CC patients. The relationship between glycolysis and EMT in CC requires further research.

### Tumor immunity microenvironment between the low- and high-risk groups

**Figure 6:**
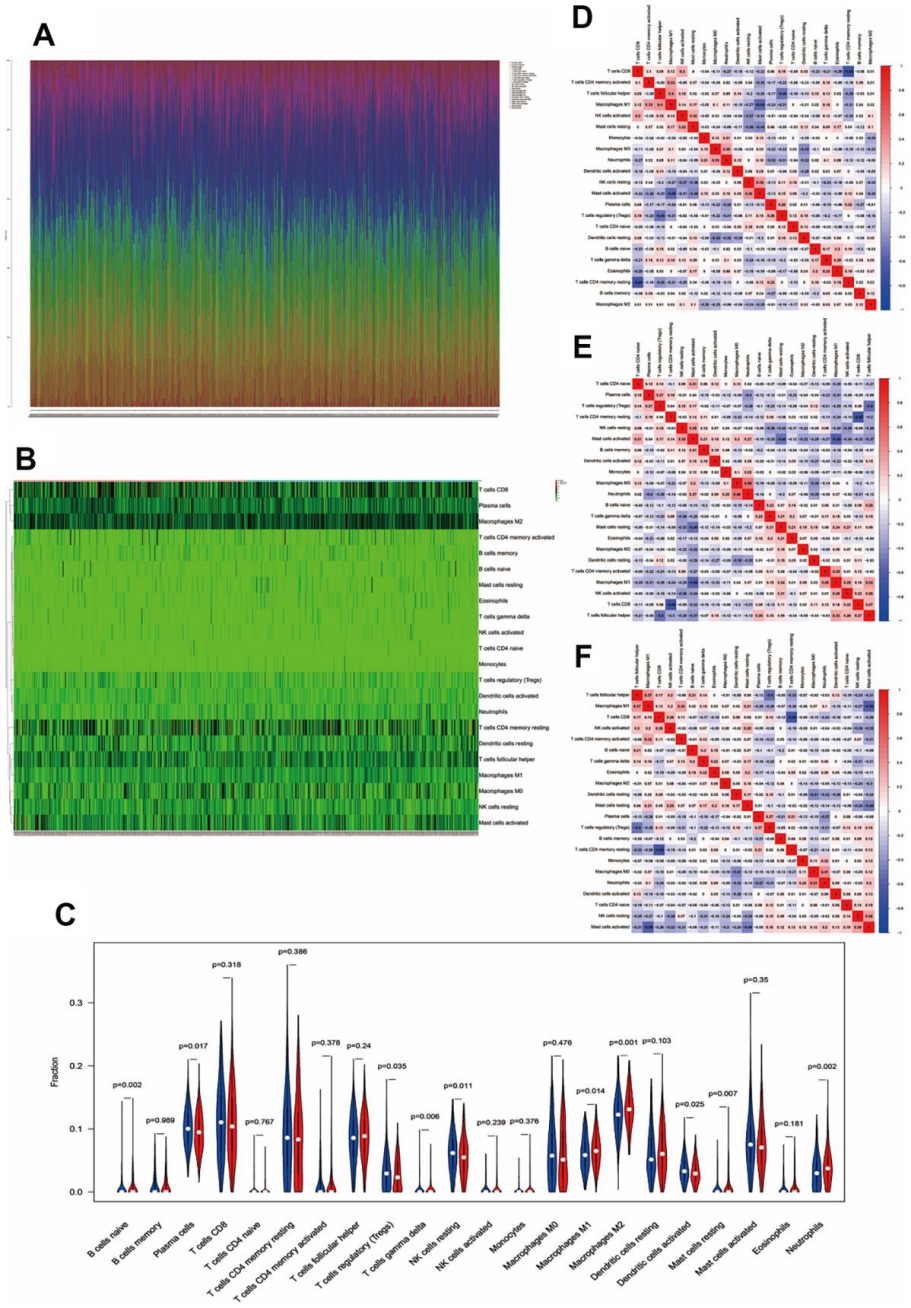
**Immune infiltration between the low- and high-risk groups.** (**A**) Fractions of immune cells in every single CC sample. (**B**, **C**) Heatmap and vioplot of immune cells between the low- and high-risk groups. Correlation between immune cells in low-risk samples (**D**), high-risk samples (**E**), and all colon cancer samples (**F**).

We investigated the fractions of 22 types of infiltrated immune cells in every single cancer among all 561 CC samples (Figure 6A). The results showed that every sample had its own immune cell infiltration characteristics. The heatmap and vioplot of infiltrated immune cells were performed between the low- and high-risk groups according to the glycolysis-related gene model (Figure 6B, 6C). In low-risk group tissues, the top five most abundant fractions of infiltrating immune cells were M2 macrophages, CD8 T cells, plasma cells, follicular helper T cells, and resting memory CD4 T cells, and this was also true for the high-risk group (Figure 6B, 6C). However, no significant fractions of memory B cells, CD8 T cells, naïve CD T cells, resting CD4 memory T cells, activated CD4 memory T cells, follicular helper T cells, activated NK cells, monocytes, M0 macrophages, resting dendritic cells, activated mast cells, or eosinophils were observed in either the low- or high-risk group. (P > 0.05, Figure 6C). Naïve B cells, M1 macrophages, M2 macrophages, resting mast cells, and neutrophils were significantly higher in the high-risk group than in the low-risk group (p < 0.05, Figure 6C). Plasma cells, Tregs, gamma delta T cells, resting NK cells, and activated dendritic cells were higher in the low-risk group (p < 0.05, Figure 6C).

Finally, the correlation of immune cells in CC tissues in both the low- and high-risk groups was analyzed. In the low-risk group, a moderate to high negative correlation was observed between the T cells CD4 memory resting and T cells CD8, M1 macrophages and activated mast cells, T cells follicular helper and Tregs, activated mast cells and resting mast cells, and resting dendritic cells and M0 macrophages; however, a positive correlation was observed between the T cells follicular helper and M1 macrophages, activated T cells CD4 memory and M1 macrophages, T cells CD8 and activated NK cells, activated NK cells and resting mast cells, resting NK cells and activated mast cells, and M0 macrophages and neutrophils (Figure 6D). In the high-risk group, a moderate to high negative correlation was observed between the resting memory CD4 T cells and CD8 T cells, M1 macrophages and activated mast cells, follicular helper T cells and Tregs, resting mast cells and activated mast cells, and neutrophils and plasm cells. However, a naïve positive correlation was observed between neutrophils and M0 macrophages, resting NK cells and activated mast cells, follicular helper T cells and M1 macrophages, activated M1 macrophages and CD4 memory T cells, and activated mast cells and CD4 T cells (Figure 6E). A negative correlation was observed between resting memory CD4 T cells and CD8 T cells, M1 macrophages and activated mast cells, follicular helper T cells and Tregs, resting mast cells and activated mast cells, and resting dendritic cells and M0 macrophages. However, a positive relationship was observed in all CC patients between M1 macrophages and follicular helper T cells, M1 macrophages and activated memory CD4 T cells, activated mast cells and resting NK cells, CD8 T cells and activated NK cells, and activated NK cells and resting mast cells (Figure 6F). In the low-risk group, high-risk group, and all cancer samples, a negative correlation was observed between resting memory CD4 T cells and CD8 T cells, M1 macrophages and activated mast cells, follicular helper T cells and Tregs, and activated mast cells and resting mast cells; however, a positive correlation was observed between follicular helper T cells and M1 macrophages and activated memory CD4 T cells and M1 macrophages.

### Validation of glycolysis-related gene prognostic prediction model in TCGA dataset

**Figure 7:**
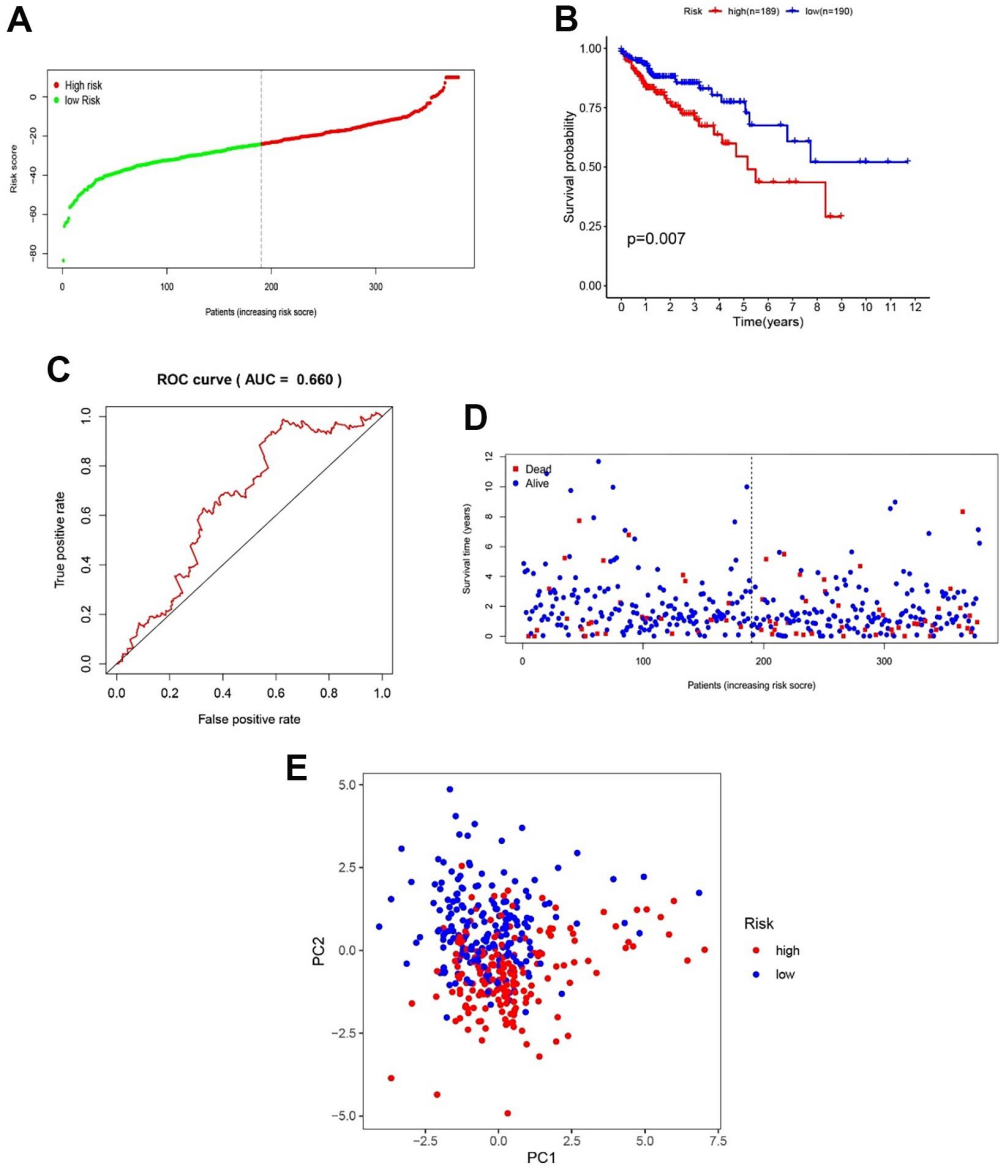
**Validation of the efficacy of the gene model in TCGA samples.** (**A**) CC patient distribution according to the median risk score. (**B**) Kaplan–Meier analysis between the low- and high-risk groups. (**C**) ROC analysis of the risk score. (**D**) The relationship between patient survival status and risk score. (**E**) PCA between the low- and high-risk groups.

RNA-seq and corresponding clinical data of 437 colon cancer patients (Tumor=398, Normal=39) were downloaded from TCGA GDC (https://portal.gdc.cancer.gov/). Matching with clinical information, 189 patients and 190 patients were classified into high- and low-risk groups according to the above glycolysis-related gene prognostic prediction model, respectively (Figure 7A). The high-risk group had a significantly poorer prognosis than the low-risk group by KM analysis (P < 0.05, Figure 7B). Then, ROC analysis showed that the AUC of our model was 0.660 (Figure 7C). The risk plot showed that a higher risk score was related to poor prognosis (Figure 7D). Our model could significantly distinguish high- and low-risk group samples through PCA (Figure 7E).

**Figure 8:**
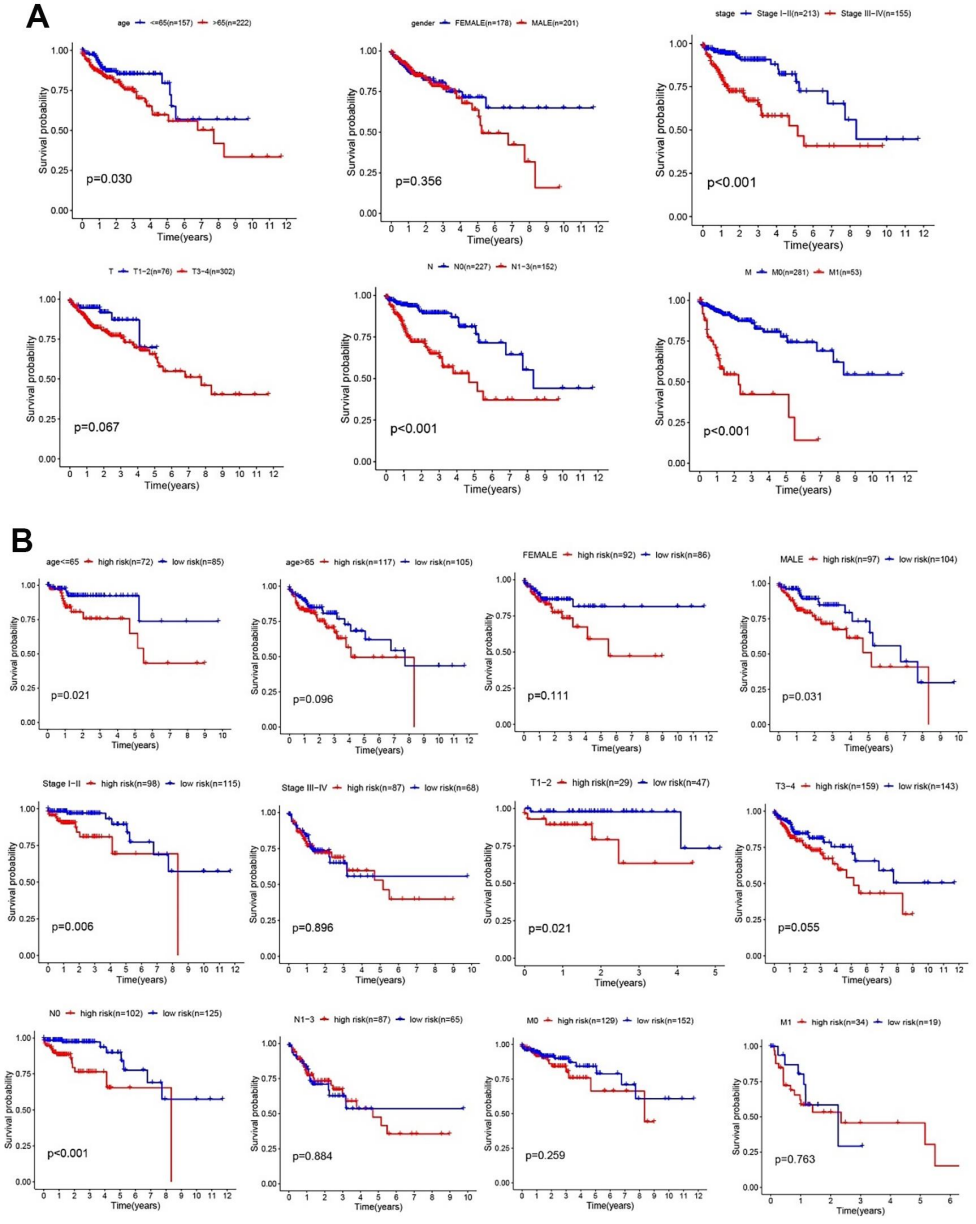
**Clinical features and risk factors in the validation cohort.** (**A**) Kaplan–Meier analysis of clinical features, including age, sex, TNM stage, T stage, N stage, and M stage. (**B**) Kaplan–Meier analysis of clinical features in low- and high-risk subgroups.

These results indicated that our gene model also had good efficacy in the TCGA validation dataset. Finally, the relationship between clinical characteristics and patient OS was examined. The results revealed that an age of > 65 years, M1, N1-3 and Stage III-IV were positively related to poor OS in the validation dataset (P < 0.05, Figure 8A). Then, the relationship between patients’ clinical features and OS in the low- and high-risk groups was analyzed, and the results indicated that high-risk patients were positively associated with poor OS when age ≤ 65 years, male sex, TNM stage I-II, T1-2 and N0 subgroups (P < 0.05, Figure 8B). These results showed that our genetic model could distinguish some clinical features and showed good efficacy. Although not all clinical features could be distinguished, this may be because TCGA colon cancer data were not as rich as GSE39582. It is also a vital reason that we used the GSE39582 dataset as a training cohort to construct the glycolysis-related gene prognostic prediction model.

### Tumor immune sensitive drug prediction

**Figure 9:**
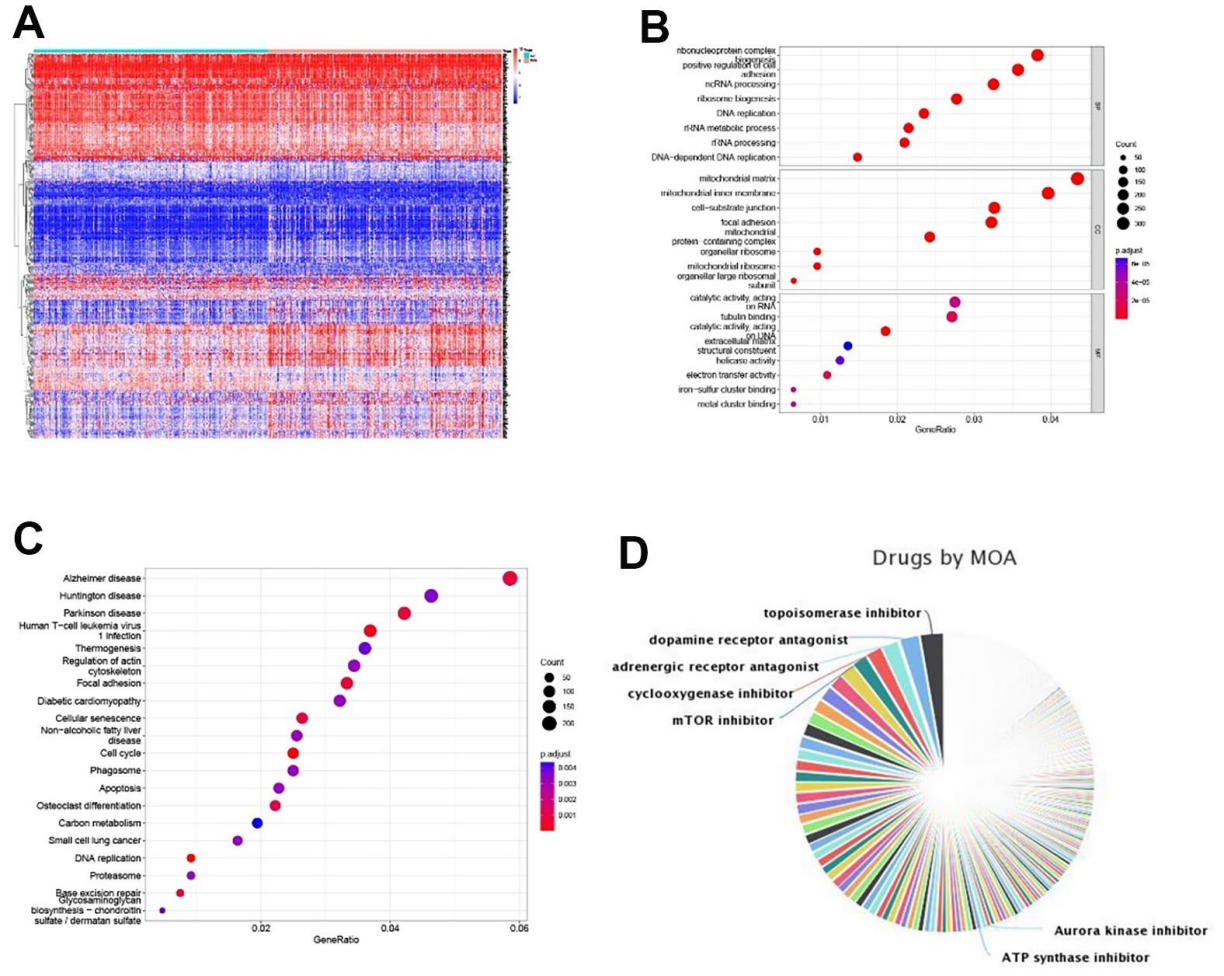
**Tumor immune sensitive drug prediction.** (**A**) Top 200 up- and downregulated significantly expressed genes between the high- and low-risk groups. (**B**, **C**) GO and KEGG enrichment analyses of differentially expressed genes. (**D**) Pie chart of tumor immune-sensitive drug types.

These studies have confirmed that glycolysis is closely related to colon cancer immunity. To further study colon cancer-sensitive tumor immune drugs in the glycolytic microenvironment, the top 400 genes of the absolute value of significantly differential expression multiples between the high- and low-risk groups were screened (P < 0.05, Figure 9A). GO function analysis of the differentially expressed genes suggested that the functions of the differentially expressed genes were enriched in DNA replication, rRNA metabolic process, mitochondrial matrix, mitochondrial inner membrane, catalytic activity and acting on RNA and tubulin binding, which are related to cell metabolism (P < 0.05, Figure 9B). The KEGG results demonstrated that the differentially expressed genes were enriched in metabolism-related pathways such as carbon metabolism, the cell cycle, apoptosis, DNA replication and the proteome (P < 0.05, Figure 9C). We screened tumor immune-related drugs through Dreimt [7], and the top 400 genes were input into the software.

The results showed that the top 3 drug types were topoisomerase inhibitors, dopamine receptor antagonists and regenerative receptor antagonists (Figure 9D). The drugs were further screened (tau absolute value > 90, top 15 of drug specificity score > 0.7), and pirarubicin/mitoxantrone (topoisomerase inhibitor), daunorubicin (topoisomerase inhibitor, RNA synthesis inhibitor), BMS-387032/CGP-60474/JNJ-7706621/AT-7519/alvocidib/purvalanol-a/PHA-793887/AZD-5438 (CDK inhibitor), bisindolylmaleimide-ix (PKC inhibitor), dactinomycin RNA (polymerase inhibitor), fludarabine (ribonucleotide reductase inhibitor) and PF-562271 (focal adhesion kinase inhibitor) were filtered out. Our data provide a theoretical basis for tumor immune targeted therapy related to glycolysis in patients with colorectal cancer.

### Validation of model gene expression in CC patient samples

To lay the foundation for the next step of our basic research, we validated the expression of all the model genes through western blotting. Our results showed that *NUP107*, *SEC13*, *ALDH7A1*, *ALG1*, *CHPF*, *GALK1*, *XYLT2*, and *OGDHL* were highly expressed, whereas *FAM162A*, *FBP2*, *IDH1*, *TGFA*, and *VLDLR* were expressed at low levels in CC patients (P < 0.05, Figure 10). These gene expression levels were consistent with the gene expression in GSE39582.

**Figure 10:**
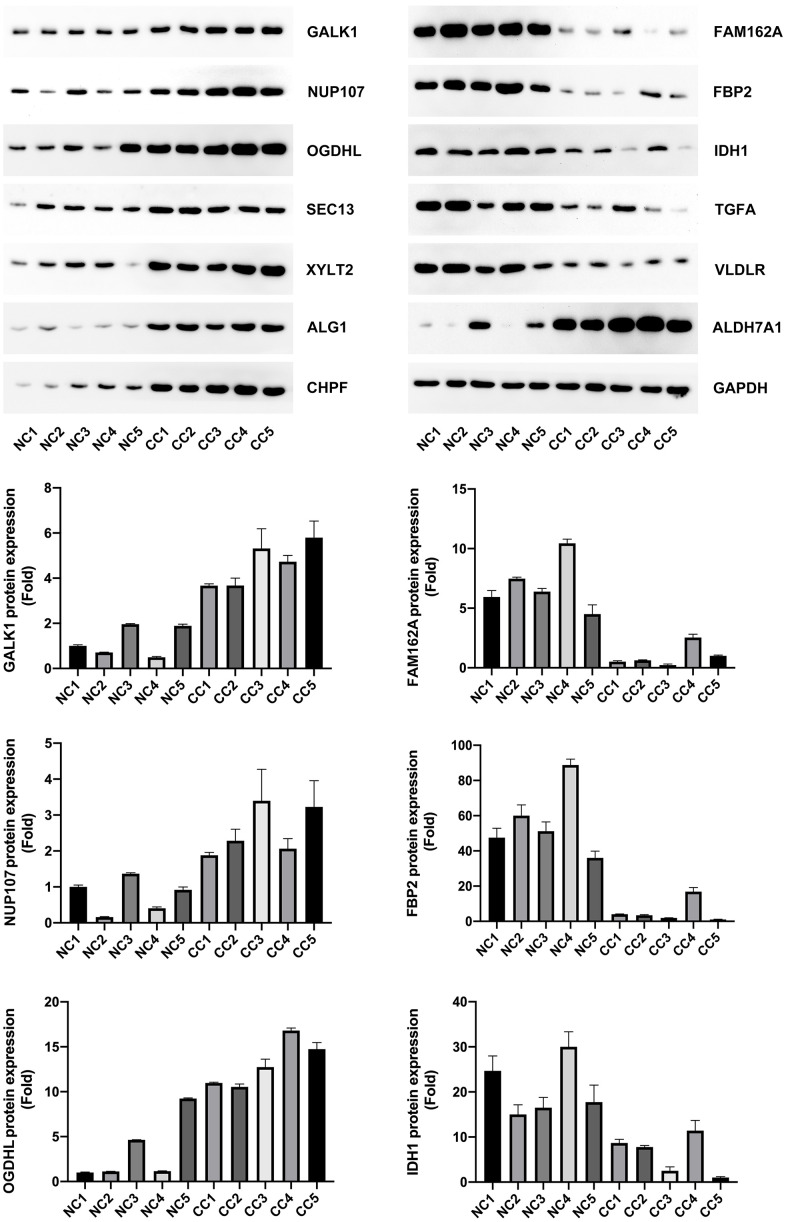
Model gene expression confirmed using western blotting.

**Figure 10:**
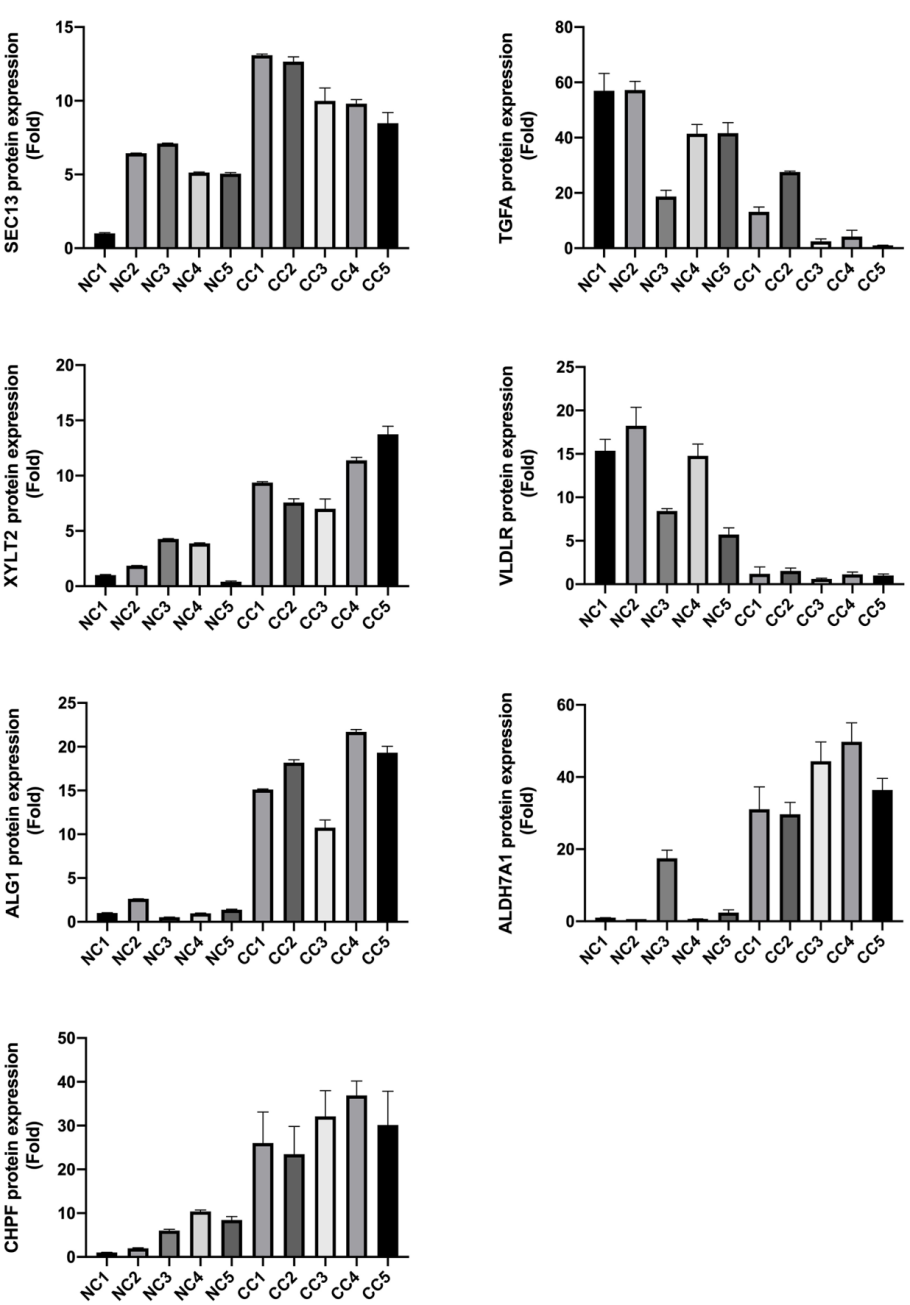
Model gene expression confirmed using western blotting.

## DISCUSSION

CC is a common gastrointestinal carcinoma that affects human health and was responsible for 1,148,515 new cases and 576,858 deaths globally in 2020 [8]. In recent years, there have been several advancements in the treatment of CC patients, including surgery, radiation, chemotherapy, adjuvant chemotherapy, and immunotherapy; however, the mortality of CC patients remains high [8, 9]. Thus, it is necessary to discover novel biomarkers as potential therapeutic targets to improve the prognosis of CC. Glycolysis is highly activated in tumor cells, and increasing evidence has revealed that reprogramming of glycolysis metabolism is closely related to the occurrence and development of tumors [10].

In this study, we collected six glycolysis-related gene sets from the GSEA database, from which we selected a total of 301 mRNAs in 566 CC patients for further analysis. Then, 217 differentially expressed glycolysis-related genes were screened for the construction of a prognostic gene model. After Cox regression analyses, a signature of 13 glycolysis-related genes (*NUP107*, *SEC13*, *ALDH7A1*, *ALG1*, *CHPF*, *FAM162A*, *FBP2*, *GALK1*, *IDH1*, *TGFA*, *VLDLR*, *XYLT2*, and *OGDHL*), all of which were independent risk factors for OS, was established between CC patient and normal tissues. This signature may be considered as a novel potential therapeutic target. Previous studies have reported that *NUP37* promotes lung cancer cell proliferation and inhibits apoptosis [11], while the overexpression of *TGFA* and *HPF* induces cell proliferation and invasion in lung cancer [11, 12]. The downregulation of *ALDH7A1* increases cell migration and invasiveness in hepatocellular and renal clear carcinomas [12]. Furthermore, *FBP2* overexpression inhibits sarcoma cell growth [13], *VLDLR* overexpression inhibits CC cell proliferation and migration [14], *OGDHL* silencing contributes to the survival of HCC patients via the regulation of glutamine metabolic pathways [15], and *IDH1* mutation promotes glioma cell proliferation and migration via EMT [16]. However, the specific roles of *SEC13*, *ALG1*, *FAM162A*, *GALK1*, and *XYLT2* in cancer remain unclear.

The prediction capability of the model was assessed through PCA, Kaplan–Meier curve analysis, and ROC analysis. The clinical variables and risk scores were also analyzed. The PCA results showed that our model specifically divided samples into low- and high-risk groups based on the risk score. Kaplan–Meier curve analysis revealed that a high-risk score was related to poorer patient prognosis. The expression of the signature genes was validated via PCR and western blotting, and the results were consistent with the gene expression in GSE39582. These results suggest that our model might be useful for predicting the prognosis of patients with CC based on the risk score. Our gene expression signature might serve as a workable tool for the classification of CC patients and may provide the basis for personalized treatment.

Among the clinical variables, age > 65 years, TNM III–IV, T3-4, N1-3, M1, and a high-risk score were identified as independent prognostic risk factors. To evaluate the clinical efficacy of the model, the relationships between the risk score and clinical features were analyzed. We found that high-risk scores in the age ≤ 65 years, age> 65 years, female sex, male sex, TNM I-II, TNM III-IV, T1-2, T3-4, M0, M1, N0, and N1-3 subgroups were highly correlated with poor OS in patients. These results established the reliability of the gene model for CC. Although not all clinical features could be distinguished in the TCGA dataset, it may be because TCGA colon cancer data were not as rich as GSE39582, which was the vital reason that we used the GSE39582 dataset to construct our prediction model. Moreover, our glycolysis-related gene signatures could clearly distinguish the tumor stage and provide the basis for potential early individualized treatment.

A study reported that EMT promotes CC cell invasion and migration and is involved in the invasion and metastasis of CC [17]. However, glycolysis is closely related to EMT in cancers [5]. In this study, the risk score was positively associated with the expression of EMT biomarkers (SNAI1, SNAI2, TWIST1, TWIST2, and ZEB2). These results suggest that high glycolysis metabolism is accompanied by changes in other regulatory pathways in the tumor microenvironment.

A previous study reported differences in immune infiltration between CC and normal tissues [18]; however, the role of tumor immunity in colon cancer glycolysis remains unclear. Our data showed that M1 macrophages and plasma cells were more and less infiltrated in the high-risk group, respectively; however, Ge et al. reported that M1 macrophages and plasma cells infiltrated significantly more in CC than in normal tissues [18]. Through immune cell correlation analysis, a moderate to high negative correlation was observed between the M1 macrophages and activated mast cells and the activated mast cells and resting mast cells in the high-risk group, low-risk group, and all cancer samples. Moreover, a positive correlation was observed between follicular helper T cells and M1 macrophages and activated memory CD4 T cells and M1 macrophages, which is consistent with the results of Ge et al. in CC compared to normal tissues. According to the above results, M1 macrophages could be a potential target for therapy and may play an important role in CC and CC glycolysis; however, the specific mechanisms remain unknown and need further research. Then, tumor immune-sensitive drugs were predicted, and pirarubicin/methoxantrone (topoisomerase inhibitor), daunorubicin (topoisomerase inhibitor, RNA synthesis inhibitor), and fludarabine (ribonucleotide reductase inhibitor) were approved for use in related clinical cancer patients. Our data provide a theoretical basis for tumor immune targeted therapy related to glycolysis in patients with colorectal cancer.

In summary, we identified and validated a prognostic model based on 13 glycolysis-related genes, including *NUP107*, *SEC13*, *ALDH7A1*, *ALG1*, *CHPF*, *FAM162A*, *FBP2*, *GALK1*, *IDH1*, *TGFA*, *VLDLR*, *XYLT2*, and *OGDHL*, for CC. This model could be positively related to poor prognosis in CC, EMT activation, and CC microenvironment immunity. The final related tumor immune-sensitive drugs were filtered. These findings could be used for the prognosis of CC patients and identifying novel biomarkers for CC therapy.

## MATERIALS AND METHODS

### Patient and control data

The Affymetrix Human Genome U133 Plus 2.0 array gene expression profile and corresponding clinical information data of 566 CC patients and 19 normal control tissues were downloaded from a GEO dataset (GSE39582). The clinical information included survival time, survival status, sex, age, tumor-node-metastasis (TNM) stage, clinical T stage, clinical N stage, and clinical M stage.

### Identification of differentially expressed glycolysis-related genes

A total of 301 mRNAs of six glycolysis-related gene sets were selected from the GSEA database (WP_COMPUTATIONAL_MODEL_OF_AEROBIC_GLYCOLYSIS, WP_GLYCOLYSIS_IN_SENESCENCE, REACTOME_REGULATION_OF_GLYCOLYSIS_BY_FRUCTOSE_2_6_BISPHOSPHATE_METABOLISM, REACTOME_GLYCOLYSIS, HALLMARK_GLYCOLYSIS, and GO_GLYCOLYTIC_PROCESS). The Wilcoxon test was used to determine the differentially expressed genes. Then, a prognostic gene model was constructed based on the 217 differentially expressed glycolysis-related genes. The cutoffs were set as Log2(fold change) > 1 and p value< 0.05. Heatmaps were generated using the Pheatmap package.

### Construction of a glycolysis-related gene risk model

Based on the 217 differentially expressed glycolysis-related genes in GSE39582, uniCox and multiCox analyses were conducted to identify the model genes. The risk score was obtained from the results of multiplication of the Cox coefficient of multiCox analysis and gene expression values. The following formula was used for the calculation of the risk score: Risk score = (Cox coefficient of model gene A* A gene expression + Cox coefficient of model gene B* B gene expression+……). Then, all patients were divided into low- and high-risk subgroups based on the median risk score. Cbioportal (http://www.cbioportal.org/) was used to screen the model gene mutation status and gene mutation sites. Survival and survminer packages were used for survival analysis. Receiver operating characteristic (ROC) analysis was performed using the survival ROC package. An area under the curve value of > 0.65 was regarded as an acceptable cutoff value. The risk score, principal component analysis (PCA), and survival status plots were generated to establish a prediction model between the low- and high-risk groups.

### Independent prognostic value of risk score

To assess the prognostic value of the model, we used univariate and multivariate Cox regression analyses to determine the prognostic factors. The factors (age, sex, TNM stage, and risk score) with p values less than 0.05 in univariate and multivariate Cox regression analyses were considered as independent factors.

### Clinical application of the gene model

To examine the efficiency of our model in CC, we analyzed the correlation between the gene model and clinical features (age, sex, clinical TNM stage, clinical T stage, clinical N stage, and clinical M stage). All patients were classified into two subgroups based on age (> 65 and ≤ 65 years old), sex (female and male), TNM stage (TNM stage I-II and TNM stage III-IV), clinical T stage (T1-2 and T3-4), clinical M stage (M0 and M1), and clinical N stage (N0 and N1-3). Survival analyses were performed between the two groups.

### Immune cell infiltration between low- and high-risk patients

Subsequently, CIBERSORT, including 22 immune cells (seven kinds of T cells, naïve B cells, memory B cells, plasma cells, resting NK cells, activated NK cells, monocytes, M0–M2 macrophages, resting dendritic cells, activated dendritic cells, resting mast cells, activated mast cells, eosinophils, and neutrophils), was used for immune cell infiltration evaluation.

### Functional enrichment analyses between low- and high-risk groups

Gene ontology (GO) analysis and Kyoto Encyclopedia of Genes and Genomes (KEGG) analyses were conducted using the org.Hs.e.g..db R package and clusterProfiler R package. A P value of < 0.05 was identified as statistically significant.

### Model validation using western blot analysis

CC patient and control tissues were collected from the Affiliated Kunshan Hospital of Jiangsu University. The patient tissues were preserved in liquid nitrogen and lysed using RIPA lysis buffer (Strong) (MedChemExpress, China). A BCA Protein Quantification Kit (Vazyme, China) was used to measure the protein concentration. Proteins were transferred to nitrocellulose membranes (Millipore, Billerica, MA, USA) after separation through 10% sodium dodecyl sulfate–polyacrylamide gel electrophoresis. Then, the membranes were incubated with primary antibodies against the following: NUP107/SEC13/ALDH7A1/ALG1/CHPF/FAM162A/FBP2/GALK1/IDH1/TGFA/VLDLR/XYLT2/OGDHL (Abcam, USA, 1:1000) and GAPDH (Abcam, USA, 1:3000). The membranes were washed and incubated with horseradish peroxidase-labeled goat anti-rabbit IgG (1:5000; Sigma). The signals were visualized using BIO-RADXR.

### Statistical analysis

Statistical analysis was performed using SPSS 21.0 software (SPSS, Chicago, IL, USA). Data were presented as the means ± standard deviation (SD). The difference between the groups was determined using ANOVA with repeated measures. To compare the difference between the two groups, the independent sample *t test* was used. P<0.05 was considered as significant.
